# PDGF-BB Does Not Accelerate Healing in Diabetic Mice with Splinted Skin Wounds

**DOI:** 10.1371/journal.pone.0104447

**Published:** 2014-08-14

**Authors:** Shin Ae Park, Vijay Krishna Raghunathan, Nihar M. Shah, Leandro Teixeira, Monica J. Motta, Jill Covert, Richard Dubielzig, Michael Schurr, Roslyn Rivkah Isseroff, Nicholas L. Abbott, Jonathan McAnulty, Christopher J. Murphy

**Affiliations:** 1 Dept. of Surgical and Radiological Sciences, School of Veterinary Medicine, University of California Davis, Davis, California, United States of America; 2 Dept. of Pathobiological Sciences, School of Veterinary Medicine, University of Wisconsin, Madison, Wisconsin, United States of America; 3 Dept. of Surgery, University of Colorado, Denver, Colorado, United States of America; 4 Dept. of Dermatology, School of Medicine, University of California Davis, Davis, California, United States of America; 5 VA Northern California Health Care System, Mather, California, United States of America; 6 Dept. of Chemical and Biological Engineering, University of Wisconsin, Madison, Wisconsin, United States of America; 7 Dept. of Surgical Sciences, School of Veterinary Medicine, University of Wisconsin, Madison, Wisconsin, United States of America; 8 Ophthalmology & Vision Science, School of Medicine. University of California Davis, Davis, California, United States of America; IDI, Istituto Dermopatico dell’Immacolata, Italy

## Abstract

Topical application of platelet-derived growth factor-BB (PDGF-BB) is considered to accelerate tissue repair of impaired chronic wounds. However, the vast literature is plagued with conflicting reports of its efficacy in animal models and this is often influenced by a wide array of experimental variables making it difficult to compare the results across the studies. To mitigate the confounding variables that influence the efficacy of topically applied PDGF-BB, we used a controlled full thickness splinted excisional wound model in db/db mice (type 2 diabetic mouse model) for our investigations. A carefully-defined silicone-splinted wound model, with reduced wound contraction, controlled splint and bandage maintenance, allowing for healing primarily by reepithelialization was employed. Two splinted 8 mm dorsal full thickness wounds were made in db/db mice. Wounds were topically treated once daily with either 3 µg PDGF-BB in 30 µl of 5% PEG-PBS vehicle or an equal volume of vehicle for 10 days. Body weights, wound contraction, wound closure, reepithelialization, collagen content, and wound bed inflammation were evaluated clinically and histopathologically. The bioactivity of PDGF-BB was confirmed by *in vitro* proliferation assay. PDGF-BB, although bioactive *in vitro,* failed to accelerate wound healing *in vivo* in the db/db mice using the splinted wound model. Considering that the predominant mechanism of wound healing in humans is by re-epeithelialization, the most appropriate model for evaluating therapeutics is one that uses splints to prevent excessive wound contraction. Here, we report that PDGF-BB does not promote wound closure by re-epithelialization in a murine splinted wound model. Our results highlight that the effects of cytoactive factors reported *in vivo* ought to be carefully interpreted with critical consideration of the wound model used.

## Introduction

Wound healing is a complex process involving coordinated interaction between cells and the extracellular matrix (ECM) mediated by cytokines and various growth factors [1]–[3]. These are harmonized by simultaneous and sequential events involving hemostasis, inflammation, cell proliferation/migration, ECM production, fibroplasia and wound contraction. Of the multiple factors that affect wound healing, the role of growth factors has been extensively studied, as they play a vital role in the regulation of cell behaviors, and elaboration and remodeling of the ECM [3]. *In vitro*, various growth factors (EGF [4], VEGF [5], [6], IGF [7], NGF [8], [9], PDGF [10]–[12]) have been demonstrated to stimulate cell proliferation, migration, and synthesis and degradation of extracellular matrix [13], [14]. *In vivo,* when applied exogenously, these factors may also promote wound healing [15]–[20]. Of the numerous cytoactive factors investigated, platelet-derived growth factor (PDGF) is the only recombinant cytokine growth factor approved by the U.S. Food and Drug Administration to promote wound closure via topical application [14]. Effects of PDGF-BB topical application to accelerate tissue repair under conditions of impaired wound healing have been demonstrated in animal models [21], [22] and human patients [23]. Currently, PDGF-BB serves as the prototypical topical growth factor to facilitate chronic wound healing [14], [22]. However, conflicting reports detailing the efficacy of PDGF exist within the vast wound healing literature [22], [24]–[27]. Especially, the models and relevant controls used in those investigations widely vary, thus making it difficult to compare the results across the studies.

Diabetes mellitus is a chronic disease that leads to impaired healing resulting in chronic wounds, for which treatment and related complications are estimated to cost $10 billion annually. The pathophysiology of diabetic wound healing and development of new agents to improve clinical outcomes are continuously being investigated. In order to understand the mechanisms involved in impaired diabetic wound healing, and to test the efficacy and safety of new therapeutic agents, many animal models have been established and utilized. In particular, murine wound models have many advantages such as relatively low cost, ease of housing and handling that in turn support larger ‘n’ studies to increase statistical power. Excisional wound models in type 2 diabetic (db/db) mice have been widely utilized for investigations of impaired wound healing including evaluating the efficacy of topical PDGF-BB applied as a single agent or in combination with other cytoactive factors [22], [24]–[27]. However, the studies of PDGF-BB on wound healing in db/db mice have resulted in inconsistent findings depending on experimental variables such as wound size, study end point, PDGF dosage, and delivery vehicle [22], [24], [26], [27]. In some reports, PDGF treatment groups, in comparison to control groups, had a decreased wound closure time or smaller final wound size [22], [24], [25], while findings from other studies suggest only an increase in granulation tissue and no significant effect on the rate of wound closure [26], [27].

Skin contracture is the primary mechanism of wound closure in rodents; therefore the appropriateness of using mice for wound healing studies has been debated [28], [29]. This is particularly important considering that re-epithelialization is an early and critical event during the wound healing process in humans [28]–[31]. To address this concern, the use of silicone splints to inhibit dermal contraction has been promoted to increase the relevancy of the murine model to wound healing in humans [30], and this model was thus selected and modified for our studies. Using the splinted wound model, it has been demonstrated that vascular endothelial growth factor accelerated wound healing in db/db mice [32].

Considering the negative to modest effect of PDGF-BB in *in vivo* wound healing literature as well as the black-box warning for incidence of tumors with prolonged application of 0.01% PDGF-BB which is the currently used in clinics, an urgent need exists to determine the appropriate clinical indication for which a cytoactive factor is used in, and corresponding animal model to test the efficacy of that cytoactive factor in chronic wound healing and in diabetic wound management. To our knowledge, however, the effect of PDGF-BB on re-epithelialization or wound closure of splinted wounds in db/db mice has not been previously published. Motivated by the conflicting results in the literature regarding the efficacy of PDGF-BB in non-splinted murine wounds, the increasing popularity of splinted wound models in assessment of therapeutic effect, and the widespread clinical use of PDGF, the study reported herein was undertaken. In this manuscript, we report a controlled approach to monitor re-epithelialization and test the efficacy of PDGF-BB in accelerating wound healing in diabetic mice. The purpose of this study was to evaluate the effect of PDGF BB on wound healing by re-epithelialization and granulation tissue formation in db/db mice using a splinted wound model. We anticipate that these data will serve as critical reference for future studies investigating various drug delivery approaches for increased cytoactive factor retention.

## Materials and Methods

### Ethics statement

All procedures adhered to the recommendations in the Guide for the Care and Use of Laboratory Animals of the National Institutes of Health. The protocol for this study was approved by the Institutional Animal Care and Use Committee of the University of California, Davis (Protocol number: 17294). All surgery was performed under isoflurane anesthesia, and all efforts were made to minimize suffering.

### Cell culture and proliferation assay

#### Cell culture

Spontaneously immortalized non-tumorigenic human keratinocyte cells (HaCaTs), generously provided by N.E. Fusenig and P. Boukamp and also catalogued with the American Type Culture Collection (ATCC; Manassas, VA), were used for this study [33]. Primary human foreskin fibroblasts or HaCaTs were routinely cultured in Ham’s F12 or DMEM medium respectively supplemented with 10% (v/v) fetal bovine serum (FBS), 1% (v/v) penicillin-streptomycin as described previously [34], [35]. Primary cells within passages 3–5 and HaCaT cells between passages 20–30 were employed for all proliferation experiments.

#### Proliferation assay

Human recombinant PDGF-BB (Shenandoah Biotechnology Inc., USA) was dissolved in phosphate buffered saline (PBS, pH 7.4) to obtain a stock solution of 100 µg/ml. This was serially diluted in serum free growth medium to obtain working concentration of 0, 7.5, 10, 50 ng/ml and 7.5 µg/ml. Cells (7000/well) were seeded in a 48 well plate in growth medium containing 1%(v/v) FBS and 1% (v/v) penicillin-streptomycin, and then treated with the various concentrations of PDGF-BB. Cell proliferation was measured after 5 days using the widely reported MTT assay [36].

#### Receptor blocking proliferation assay

Prior to seeding, cells were incubated with 10 µg/ml of goat polyclonal anti-PDGF receptor beta antibody for 30 min at 37°C on an orbital shaker. Cells were centrifuged at 1400 rpm for 5 min, the supernatant discarded and the pellet resuspended in growth medium and then plated in the presence of soluble of PDGF-BB as described above. Proliferation was determined after 5 days using the MTT assay.

### Animals

Twenty-four genetically diabetic 16 week old male mice (db/db; BKS.Cg-m +/+ *Lepr^db^*; Jackson Laboratories, Bar Harbor, ME) were used for this study. The average ± SEM body weight of the mice was 52.6±0.4 g. Mice were housed four animals per cage pre-surgery and alone post-surgery; they were maintained in an animal care facility with a 12 hour light/dark cycle for four weeks prior to surgery and thereafter. Mice were randomly assigned to one of two treatment groups: 30 µl of 0.01% (w/v) PDGF-BB in vehicle (PDGF group; n = 12) and 30 µl of vehicle control [5% (w/v) poly(ethylene) glycol (VEHICLE) group; n = 12]. The animal number was calculated with power and sample size analysis based on our previous study results. Alpha and beta levels were set at 0.05 and 0.8, respectively.

### Anesthetic and Surgical Procedure

Anesthesia was induced and maintained with 2.25–2.50% isoflurane in 100% oxygen, flow rate of 1.5–2 L/minute, using a multi circuit anesthesia system for rodents (SAA2-3, Viking medical, Minneapolis, MN). Buprenorphine HCl (0.05 mg/kg, Buprenorphine HCl Injection, Ben Venue, OH) and lactated ringer’s solution (10 mg/kg, Lactated ringer’s injection USP, Baxter, Deerfield, IL) were administered subcutaneously following induction.

The mouse was positioned in ventral recumbency on a temperature controlled heating platform (ACT 100, World precision instruments, Sarasota, FL). The dorsal surface was shaved with an electric clipper, sterilely prepped with 4% chlorhexidine and 70% alcohol gauze sponges, and then sterilely draped. All wounding procedures and post-operative treatments were performed by one surgeon. Prior to wounding, two sterile donut shaped splints (inner diameter of 10 mm, outer diameter of 14 mm) fabricated from 1.6 mm thick silicone sheet (Press-to-Seal Silicone Sheet JTR-S-2.0, Grace Bio-Labs, Bend, OR) were placed bilaterally around the pre-marked designated wound location (the center of the wounds were located 40 mm cranial from the tail head), and then adhered with an immediate-bonding cyanoacrylate adhesive (Krazy Glue, Elmer’s product, Inc., Columbus, OH) and eight interrupted sutures using 6-0 nylon sutures (6-0 Ethilon Nylon Suture, Ethicon LLC., Cornelia, GA). A full thickness wound was created inside each splint by using an 8 mm sterile skin biopsy punch and a Westcott scissor. A trimmed sterile plastic cover slip (Fischer Scientific, Pittsburgh, PA) was placed on top of the splint, and a semi-occlusive dressing (Tegaderm Film 9506 W, 3 M Health Care, St. Paul, MN) was applied circumferentially around the trunk of the animal. Animals were placed under a warming lamp and observed until they recovered fully from anesthesia.

### Clinical Evaluations and Treatments

Body weight, appetite, symptoms for pain (depression, agitation, self-mutilating, and shivering), and general health condition were monitored daily, and additional pain medication (buprenorphine HCl, 0.05 mg/kg sc) was administered as needed. Mice were briefly anesthetized with isoflurane for about 5 minutes daily during bandage replacement, wound bed assessment, imaging, and treatment. The semi-occlusive dressing was cut open daily, along the coverslips placed on top of the splints, and the wound bed was evaluated for splint stability, inflammation, or infectious exudates. If excessive fluid accumulation was found, the fluid was gently wicked away with a cotton tip applicator. Wound beds were photographed on days 0, 2, 4, 6, 8, 10, and 11, with standardized exposure and focal length using a digital camera with macro lens and ring flash (Nikon D300, Nikon, Melville, NY) mounted on our custom-made imaging station that provided incorporation of a ‘mm’ scale bar in each image obtained. Mice in the PDGF group received 30 µl of 0.01% PDGF-BB (i.e. 3 µg dosage, PDGF-BB Human Recombinant, Prospec, Ness-Ziona, Israel) dissolved in vehicle, filter-sterilized 5% PEG (P156-500 Carbowax PEG 8000, Fisher Scientific, Santa Clara, CA) in phosphate-buffered solution, while the vehicle control group received 30 µl of the PEG vehicle. Both wound beds in each animal were treated identically immediately after surgery and once daily for 10 days. Following evaluations and treatments, new sterile coverslips were placed over the splints and a piece of Tegaderm dressing (Tegaderm Film 9506 W, 3 M Health Care, St. Paul, MN) was applied circumferentially around the trunk of the mice. Bandages were replaced on days 2, 5, and 8. All mice were euthanatized with an intraperitoneal injection of Pentobarbital plus phenytoin (0.3 ml; Buthanasia-D Special, Schering-Plough Animal Health, Union, NJ) on day 11 following blood glucose measurement using a glucometer (AlphaTRAK Blood Glucose Monitoring System, Abbot Laboratories, North Chicago, IL).

### Image Analysis

**Figure 1:**
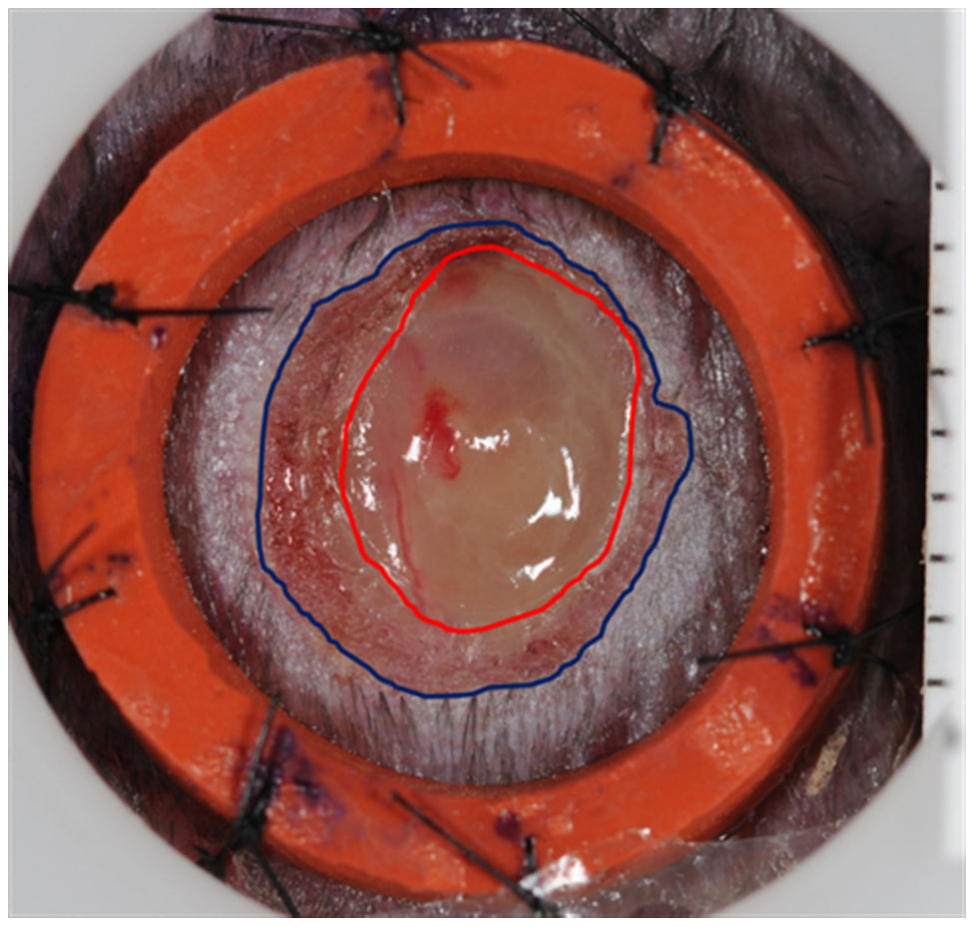
Photographs of a wound on Day 6 in a db/db mouse treated with PDGF-BB. Area wound closure was calculated as original wound area on Day 0– open wound area on Day ‘X’ (area inside inner circumference indicated by a red line). Area wound contraction was calculated as original wound area on Day 0– area including open wound and new epithelium on Day ‘X’ (area inside outer circumference indicated by a blue line).

Photographs were analyzed to calculate percentage wound closure and percentage wound contraction using image analysis software (ImageJ, National Institutes of Health, Bethesda, MD, USA) by a masked investigator. The outer and inner edges of wound closure (**Fig. 1**) were traced. The area between these edges the inwardly advancing newly epithelialized skin. Percentage wound closure at each time point was calculated as **(** **Fig. 1** **)**:

*% Wound Closure = (Wound Area on Day 0– Area of Inner Circumference on Day X)/Wound area on Day 0×100*.

Percentage wound contraction of each time point was calculated as:

*% Wound Contraction = (Wound Area on Day 0– Area of Outer Circumference on Day X)/Wound Area on Day 0×100*.

Note, the area of inner circumference excludes reepithilialization, while the area of outer circumference includes reepithelialization **(see** **Fig. 1** **)**.

### Histopathological Analysis

All histopathological analysis was performed by a board certified veterinary pathologist who was unaware of experimental group for each sample. Following euthanasia, the entire wound and unwounded skin margins greater than 5 mm were excised to the depth of the retro-peritoneum. Samples were fixed in formalin for at least 24 hours and then sectioned through the center of the lesion to obtain the largest diameter of the wound. Tissue samples then underwent routine paraffin processing, were serially sectioned at a thickness of 5 µm and stained with hematoxylin and eosin, and picrosirius red. The widest section obtained was measured. The sections were photographed using a mounted digital camera (Olympus DP72, Melville, NY) and images were analyzed using image analysis software (CellSence Dimension 1.4, Olympus, Melville, NY) for length of reepithelialization, epithelial gap, amount of fibrovascular proliferation (granulation tissue) in the dermis, and inflammatory response.

**Figure 2:**
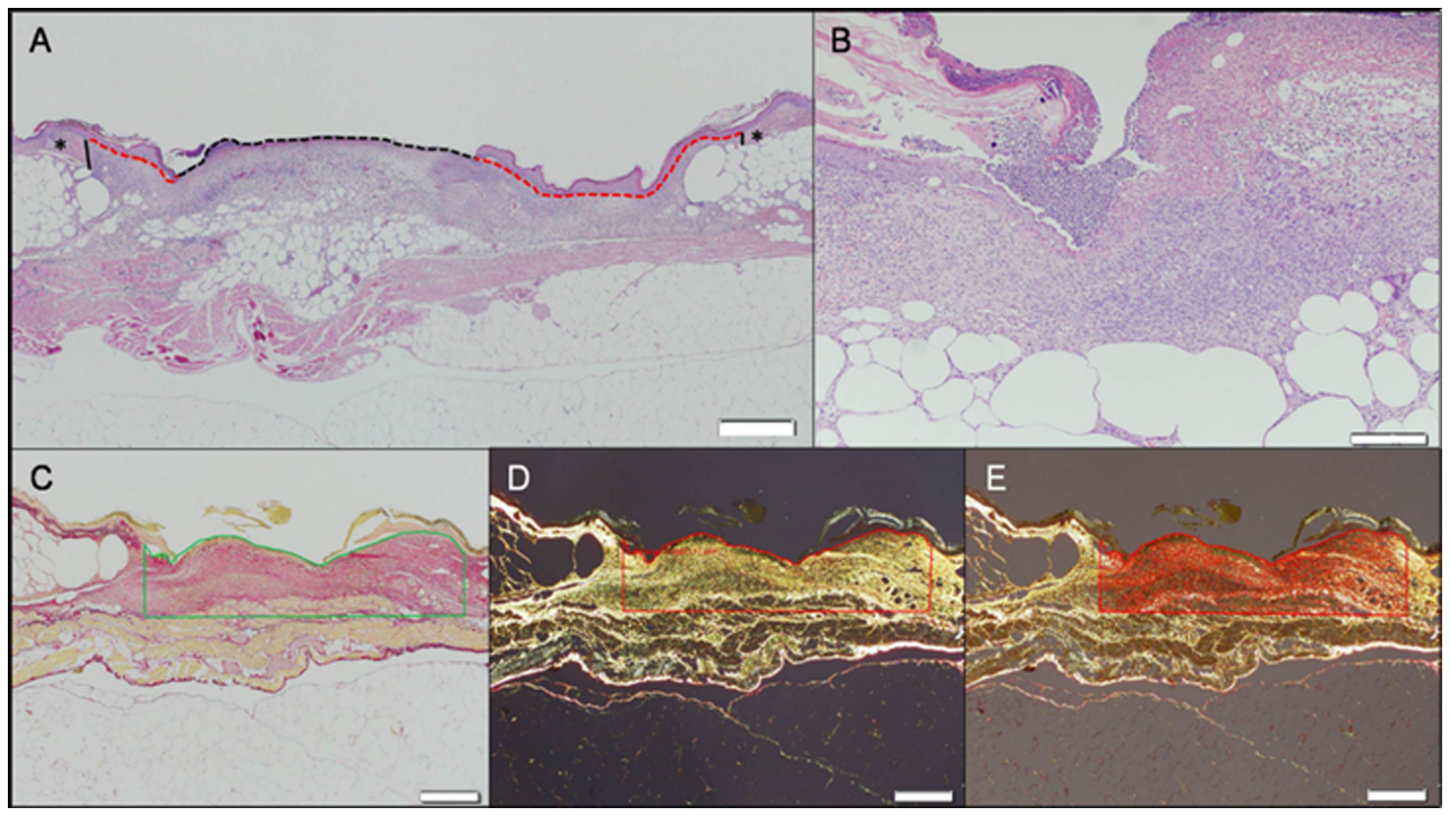
Histopathological evaluations of a wound bed from a db/db mouse treated with PDGF-BB for 10 days and harvested 11 days after wounding. Scale Bars = 500 µm. (**A**) Measurement of epithelial gap (dotted black line) and reepithelialization (dotted red lines). The epithelial gap was defined as the distance between the advancing edges of keratinocyte migration measured in millimeters. Length of reepithelialization was defined as the length of the layer of proliferating keratinocytes covering the wound area. This value was obtained by measuring s the distance between the free edge of the keratinocyte layer and the base where the cells were still associated with native, non-affected dermal tissue (*). The black vertical lines denote the area of separation between the pre-existing collagen and wound bed. The final value was the sum of distance in millimeters of both sides. H&E staining. (**B**) Inflammatory infiltrate in the wound bed. The inflammatory response was assessed using a semi-quantitative scoring system ranging from 0 to 4 where 0 indicates no inflammation, 1 indicates 0–25% of the wound area affected, 2 indicates 25–50% of the wound area affected, 3 indicates 50–75% of the wound area affected, and 4 indicates >75% of the wound area affected. This sample was classified as score 2. H&E staining. (**C, D and E**) Measurement of the fibrovascular dermal proliferation in the wound bed. Picrosirius red staining. (**C**) The wound bed area is outlined (green lines), comprising a preset depth of 0.75 mm (this is the average depth of the wounds in the experiment) and the borders between preexisting dermal collagen and newly formed collagen. (**D**) Under polarized light the bright collagen fibers of the wound bed are highlighted and automatically measured by the software (**E**), and the final data is expressed as a percentage of outlined wound area.

Length of reepithelialization was defined as the length of the layer of proliferating keratinocytes covering the wound area. This value was obtained by measuring the distance between the free edge of the keratinocyte layer and the base where the cells were still associated with native, non-affected dermal tissue **(** **Fig. 2A** **)**. Both sides of the lesion were measured and the final result was the sum of the two measurements. For wounds that had been completely re-epithelialized, a single measurement was taken from dermal edge to dermal edge. The epithelial gap was defined as the distance between the advancing edges of keratinocyte migration **(** **Fig. 2A** **)**. Fibrovascular dermal proliferation (granulation tissue) was measured by examining the Picrosirius red-stained sections under polarized light which highlighted the newly deposited dermal collagen. Using the image analysis software the wound bed area was selected and the amount of collagen deposition in the selected area was automatically measured and was expressed as a percentage of the wound area **(** **Fig. 2C, D and E** **)**. The inflammatory response was assessed using a semi-quantitative scoring system ranging from 0 to 4 where 0 indicates no inflammation, 1 indicates that 0–25% of the wound area was infiltrated by inflammatory cells, 2 indicates 25–50%, 3 indicates 50–75%, and 4>75% of the wound area was infiltrated.

In our study, instead of subjectively estimating the area of apparent granulation tissue formation in the wound bed, we determined the collagen content in the whole wound by a semi-quantitative method. Preliminary studies verifying the validity of this approach demonstrated a correlation between a simple measurement of the area of peripheral granulation tissue with the quantitated collagen content. In our study collagen content in the wound bed was measured by quantifying the amount of Picrosirius red stained tissues under polarized light microscopy and using automated imaging analysis. We accurately determined the early fibroblastic reaction infiltrating the center of the wound in addition to the dense and well-formed granulation tissue at the edges of the wound bed. We believe that this method provides an accurate representation of the fibrovascular reaction associated with the healing process in the whole wound bed, especially in the diabetic mice where infiltration of fibrovascular tissue between the adipocytes is a feature [37].

### Exclusion Criteria

Exclusion criteria included wound infection, spontaneous death, tissue destruction during processing for histopathological examination, and splint failure. Splint failure was defined as one or more of the following: splint fracture; partial or complete detachment of splint; two or more sutures missing or released. When one missed or released suture was found during daily examination, the suture was restored.

### Statistical Analysis

Results are expressed as mean ± SEM. Data from individual mouse bilateral wounds were averaged and considered as one data point. If one of the two wounds were excluded during the experiment based on exclusionary criteria, data from only the inclusionary wound was used as the data point for comparisons. Statistical analysis was performed using a commercially available statistical software SPSS (version 15.0, SPSS Inc, Chicago, IL). For the *in vitro* proliferation assay, statistical significance was determined by one-way analysis of variance (ANOVA) followed by Dunnett’s multiple comparison test. The effects of mouse strain, presence of the splint, dressing, treatment, time, and interaction between time and each effect on the body weight, wound healing, and contraction were evaluated using repeated measures ANOVA. Histological scores were compared between the groups by using the Student’s t-test. Values of P<0.05 were considered significant.

## Results

### PDGF-BB stimulates cell proliferation *in vitro*

**Figure 3:**
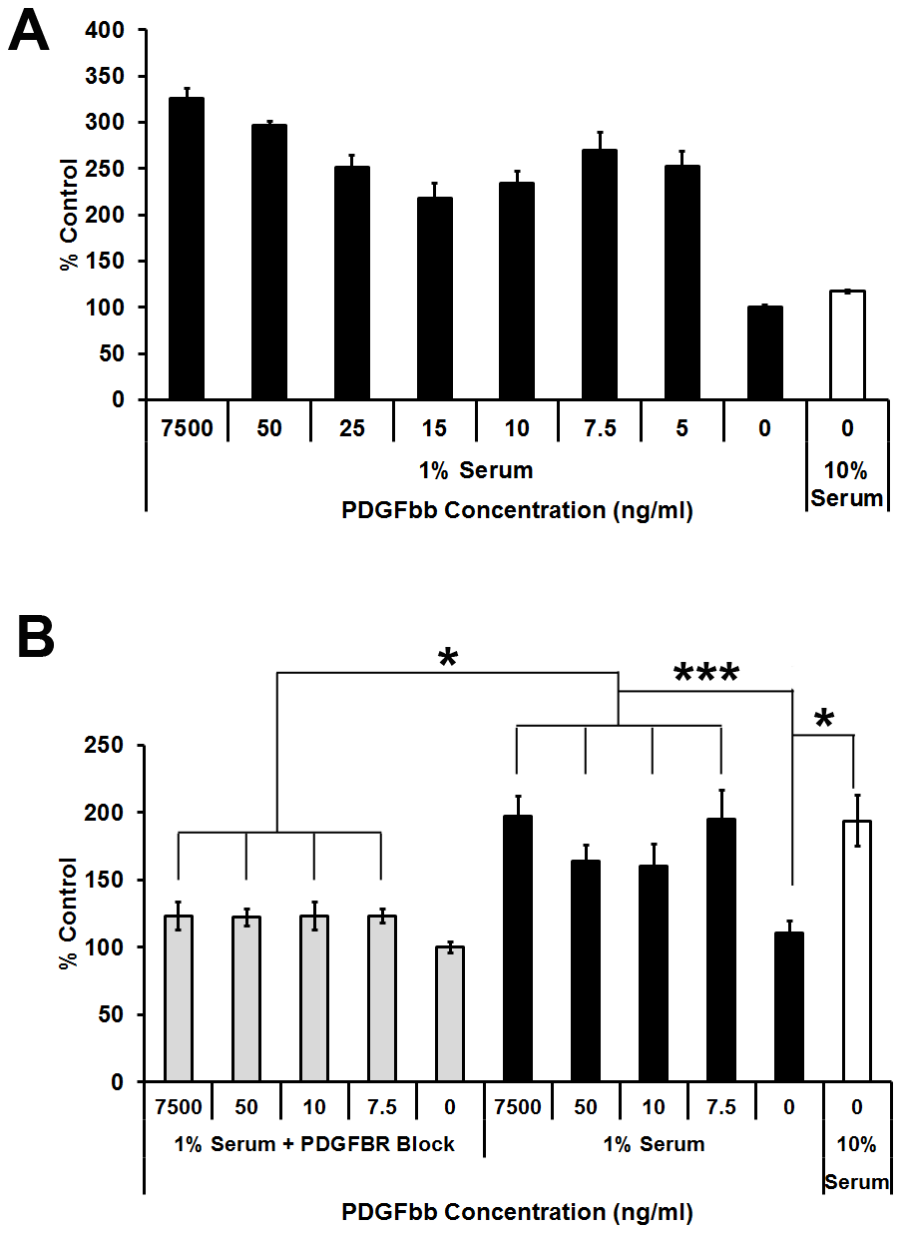
Soluble PDGF-BB promotes proliferation of primary foreskin fibroblasts over 5 days, and can be attenuated by blocking PDGF beta receptors. (**A**) A trimodal dose dependent response was observed with peak proliferation at 7.5 ng/ml, 7.5 and 50 µg/ml. Results are Mean ± StDev, n = 6. (**B**) Cell proliferation over 5 days was inhibited for all concentrations of PDGF-BB when PDGF-B receptor in cells were blocked with 10 µg/ml of PDGF beta receptor antibody for 30 min at 37°C prior to cell seeding. Results are Mean ± StDev, n = 6. * P<0.05, *** P<0.001.

Proliferation was significantly higher in PDGF-BB treated fibroblasts (all doses) compared to untreated cells. The dose response was trimodal in nature. Maximum proliferation was observed for the following doses: 7.5 ng/ml, 50 ng/ml and 7.5 µg/ml **(** **Fig. 3A** **)**. Proliferation in treated cells, while remaining higher than in untreated cells, appeared to decrease between 7.5 ng/ml to 15 ng/ml before again increasing. When the cells were blocked with an antibody to the PDGF beta receptor, proliferation was significantly reduced for all concentrations, approximating results obtained for untreated cells **(** **Fig. 3B** **)**. HaCaT cells treated with PDGF-BB did not show any demonstrable significance in proliferation rates (data not shown).

### PDGF-BB does not promote closure of splinted wounds in db/db mice

**Figure 4:**
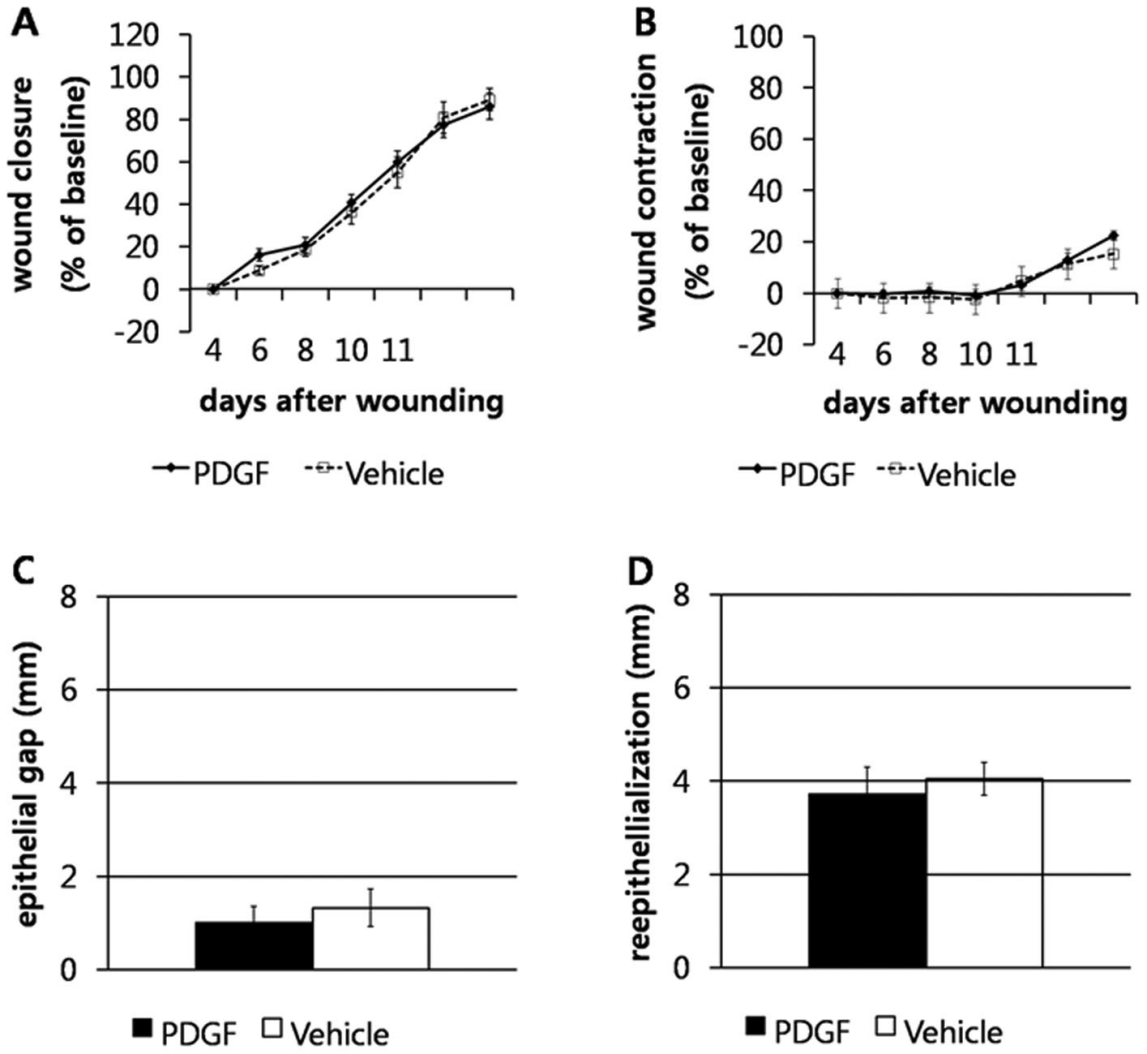
PDGF-BB did not accelerate wound closure, contraction or reepithelialization in the mouse splinted full thickness dermal wound model. Percentage wound closure (**A**) and contraction (**B**) as mean ± SEM in full thickness splinted wounds of db/db mice treated with 0.01% PDGF-BB or vehicle control. Wound area was measured from photographs taken immediately after wounding, and then the area including open wound plus new epithelium was measured from photographs taken day 2, 4, 6, 8, 10, and 11. Percentage wound contraction and closure was calculated as described in methods. Epithelial gap (**C**) and reepithelialization (**D**) were measured by histopathological evaluation. All data were expressed as mean ± SEM. No significant differences between groups were found in any comparision.

**Figure 5:**
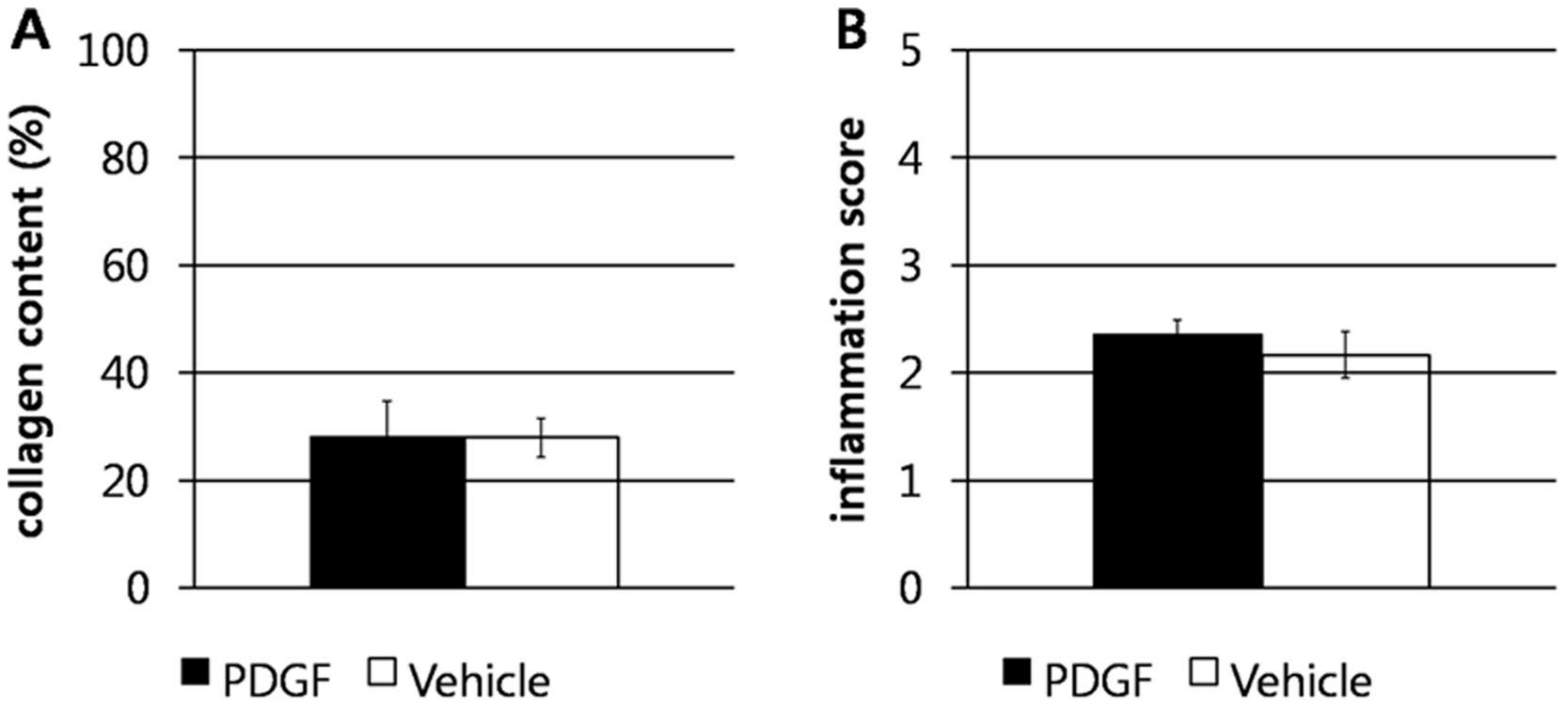
PDGF-BB did not modulate collagen deposition and inflammation development at the wound site in the splinted full thickness dermal wound model in mice. Percentage collagen content (**A**) and inflammation score (**B**) as mean ± SEM in full thickness splinted wounds of db/db mice treated with 0.01% PDGF-BB or vehicle control. No significant differences in percentage collagen content or inflammation score were found (P>0.62).

Unilateral wound from 8 mice (5 controls and 3 treated) were excluded from the study based on our strict exclusion criteria. However we were able to use data obtained from the contra-lateral wounds, therefore all animals (24/24) were included for data analysis. Mean ± SEM percentage wound closure on day 11 was 86.1±6.2% in the PDGF group and 89.7±5.3% in the vehicle group **(** **Fig. 4A** **)**. Mean ± SEM percentage wound contraction was 22.6±1.9% in the PDGF group, and 22.0±4.7% in the vehicle group **(** **Fig. 4B** **)**. In overall comparisons, no significant effect of treatment on wound contraction or closure between the PDGF and VEHICLE groups was observed **(** **Fig. 4** **A, B)**. In histopathological evaluations, there were no significant differences in epithelial gap (P = 0.74) or reepithelialization (P = 0.47) (**Fig. 4** **C, D**). There were also no significant differences in collagen content (P = 0.62) or inflammation score (P = 1.00, **Fig. 5**) between groups.

## Discussion

In this study, the applicability of a splinted wound model to ascertain the ability of PDGF-BB in facilitating wound closure was evaluated. The results of this study would become baseline data for using the splinted wound model to evaluate advanced technologies developed to improve delivery of PDGF-BB for the treatment of chronic wounds.

The bioactivity and specificity of PDGF-BB in facilitating cell proliferation was determined using *in vitro* cell proliferation assays. *In vitro,* PDGF-BB significantly accelerated cell proliferation in a trimodal pattern with maximum effects observed at 7.5 µg, 50 ng and 7.5 ng in this study **(** **Fig. 3A** **)**. Blocking PDGF receptor beta significantly inhibited the proliferative ability PDGF-BB. *In vivo*, significantly accelerated wound closure was demonstrated in previous studies [25], [38], [39] when PDGF-BB was applied in the order of micrograms per wound. Based on these previous studies and results from our *in vitro* studies and, a dose of 3 µg per wound (8 mm diameter) was chosen for this study, which corresponds to approximately 6 µg/cm^2^. Wounds were treated daily for 10 days after wounding. Considering that the wounds in our study were smaller (at least one fourth the wound area compared to other studies) we chose to monitor wound closure over 11 days after wounding instead of the 21 days for the larger wounds previously reported [25], [38], [39].

Previously published reports have shown variability in efficacy of PDGF-BB in promoting closure of nonsplinted wounds in db/db mice depending on experimental conditions such as PDGF dosage, treatment duration, and study endpoint [24]–[27], [38], [39]. Predominant experimental differences between previous studies [24], [25], [39] and this one were (a) wound size, which for our study was approximately four times smaller (based on total wound surface area), and (b) duration of follow-up (11 days vs 21 days). On gross image analysis, we observed that wounds in both groups (PDGF-BB and vehicle control) had approximately 85–90% closure 11 days after wounding. This endpoint is concurrent with previous studies showing similar healing rates of 80.9, 88.2, and 90.6% in PDGF treated groups on day 21 [25], [39]. Greenhalgh et al [25], the first to report that PDGF-BB significantly enhanced wound healing in full thickness wounds of db/db mice, demonstrated that effective wound closure accompanied by improved granulation tissue formation was observed 21 days after wounding in a 15 mm×15 mm square full thickness wound on the back of mice treated with 10 µg per wound PDGF-BB (4.4 µg/cm^2^ wound area) for 5 and 10 days. However, they did not observe any differences in wound closure up to 10 days after wounding for treated groups. Other studies [24], [39], [40] have monitored larger wounds for up to 21 days after wounding and have demonstrated a similar positive effect. Considering the differences in results between our study and others, it can be inferred, retrospectively, that the size of a wound accompanied by the duration of follow-up are critical parameters to consider while demonstrating any potentially observable effects of PDGF-BB.

Another difference between previous studies [24], [25], [38], [39] and this study is that we placed a silicone splint around the wound to reduce wound contraction. This is a critical difference when considering the primary mode of wound healing in rodents (by contraction) and humans (by reepithelialization). While Senter et al. [26] hypothesized that the primary effect of PDGF-BB on wound healing is by acceleration of contraction, other studies have suggested that PDGF-BB facilitates wound healing *in vivo* by chemotactic and mitogenic promotion of granulation tissue formation and reepithelialization [25], [39]. However, direct comparisons to these studies are not feasible due the variation between species, models, treatment times, wound size etc. In order to mitigate the number of unknowns, in this study, we investigated the efficacy of PDGF-BB in facilitating wound closure by re-epithelialization using a controlled, validated and well defined splinted full thickness wound model. This model was developed to minimize multiple confounding factors often affected by inconsistent splint and bandage maintenance, and direct contact of bandage to wound surfaces. Our data clearly demonstrate that there are no differences observed in wound contraction or epithelialization in splinted mouse wound model in the presence or absence of PDGF-BB. To our knowledge there are no published reports detailing the differences in PDGF-BB mediated wound contraction observed between a splinted and unsplinted model.

Another factor that can impact wound closure is the dressing methods. The importance of application and maintenance of semi-occlusive dressings, and infection control have been discussed previously [27]. A correlation between accumulation of exudate/fluid and decreased reepithelialization in db/db mice has been previously reported [26]. In our study, we placed a sterile coverslip over the splint to prevent direct contact of adhesive dressing with the wound bed; this was done to protect the wound bed and to prevent any inadvertent effects on wound healing. Additionally, excessive fluid was removed prior to treatment, and coverslips were replaced daily in order to maintain a clean wound bed. It is thus possible that by our dressing method and by maintaining a clean wound bed, a better environment for healing was provided for both control and PDGF groups.

The bioactivity of PDGF-BB used in the study as confirmed *in vitro* using fibroblasts and keratinocytes. Our *in vitro* results demonstrate that PDGF-BB did not accelerate proliferation of HaCaTs, but effectively increased proliferation of primary human foreskin fibroblasts. The failure to promote proliferation of HaCaTs in response to PDGF-BB was anticipated as they inherently lack PDGF beta receptors [41]. A prerequisite for PDGF to promote wound healing is that the cells present in the wound bed express PDGF receptors. *In vivo* the baseline expression of PDGF receptors have been reported to be significantly inhibited in genetically diabetic db/db mice; expression of the B-type receptor is further inhibited during repair [42]. Although we did not quantify the *in vivo* expression of PDGF receptors in our study, we can relate our findings and infer from previous studies that the expression of PDGF B receptors, required for a PDGF-BB specific response, is significantly inhibited. This may lead to inhibition of both the autocrine and paracrine signaling effects of PDGF required for healing. We also recognize that the expression and activities of matrix metalloproteinases and other proteases are altered during diabetes and may contribute to accelerated degradation of growth factors [43]–[46] present inherently in the wound site or those topically administered. In this study, we did not observe any impairment or acceleration in wound closure by re-epithelialization in PDGF treated mice when compared to control groups. While it is indeed possible that proteases may have led to excessive proteolysis of PDGF-BB administered, contrary to the recommended clinical usage of PDGF-BB for treatment of diabetic foot ulcers, we did not observe any change (impairment or acceleration) in wound healing (in *db/db* mice) upon PDGF treatment using the splinted model.

Advanced protein glycosylation is a process of non-enzymatic modification of proteins and plays a significant role in ageing and diabetic pathology. Advanced glycation end products (AGE) generated as a result of this process can exacerbate the disease process via oxidative damage, apoptosis and inflammation, and is implicated in Alzheimer’s disease, cardiovascular disease and diabetes mellitus to name a few [47]. Indeed, AGE precursors can impair or alter the post-translation modification of receptors of growth factors thus modifying their biological activity. For example, AGE precursors inhibit epidermal growth factor receptor (EGFR) dephosphorylation, and promote EGFR crosslinking thereby modifying EGF signaling [48]. Similarly, methylglyoxal (an AGE precursor) can induce AGE formation resulting in PDGF receptor β dysfunction [49]; also, glycation of PDGF itself can also inhibit its biological activity and may thus impair wound repair [50]. Consistent with these, we observed that treatment of PDGF-BB did not have any significant effect on wound healing. In this study, the extent to which AGE mediated inhibition of PDGF receptor or glycation of exogenously added PDGF-BB contributes to a lack of accelerated wound healing is not known.

The results of this study demonstrated no detectable effect of PDGF-BB on wound healing in db/db mice with splinted full thickness wounds. A species specific difference in the usage of human recombinant PDGF-BB to treat chronic wounds in mice is highly unlikely considering that PDGF-BB is mitogenic in murine cells [51], [52]. These results are very important to take into account while making a choice between PDGF-BB or other cytoactive factors for treatment of chronic wounds; especially considering that the use of PDGF-BB is associated with an FDA ‘black box’ warning for potential neoplastic growth. However, this study clearly identifies multiple confounding variables within an animal model while investigating the effects of cytoactive factors for wound healing. In summary, the use of a splint for excisional wounds is optimal to observe wound closure by reepithelialization. Meticulous application, maintenance and monitoring of the wound bed with minimal complications are possible with our modified silicone splinted model. This study clearly demonstrates that the effects of cytoactive factors reported *in vivo* needs to be carefully interpreted with critical consideration of the wound model used and there is much remaining to be understood about the effect of growth factors for treatment of chronic wounds, especially in disease models.
